# Putting Away Winter Clothing

**Published:** 1891-06

**Authors:** 


### PUTTING AWAY WINTER CLOTHING.

In the first place, it should not be put away too early, especially winter underclothing. Outer garments can be much more safely lightened, if only wraps are kept handy—as, indeed, they should be all summer—for driving and sudden changes of weather. Before putting garments away, let them be mended and thoroughly cleaned. Dirt invites moths; and, besides, what a satisfaction there is in taking out clothing that is all ready to put on. The moth is the bane of the housekeeper, but, after all, it is not difficult to escape its inroads. The mother moth flies about in search of a suitable place to deposit her eggs, and she selects woollen fabrics or fur, and likes it all the better if it is soiled. The grub, once out of the egg, feeds on what is nearest it, and so we find an assortment of holes where we left solid cloth. Now, if garments are put away clean, and absolutely free from moths' eggs, and are protected from the flying moths, they are safe without camphor or any of the disagreeable odors that are resorted to. Stout calico bags, sewed up with double seams, and tied tightly at the tops with tapes, are most useful. Let all be distinctly labelled, and not be so large but that each can be devoted to one class of garments. For instance, imagine the convenience of a row of bags hung up in your store-room, one labelled children's woollen stockings; another, woollen hoods, tippets, gloves, and mittens, and so on. How easy to get them the moment they are wanted, without diving to the bottom of a miscellaneously filled trunk. Coats, dresses, etc., that must not be tumbled, may be nicely folded, wrapped in newspaper, and laid in large paper boxes, labelled, and put on the closet shelf. Long bags, the full length of dress or cloak, with hanging loops at top, save from creasing, as well as from dust and moths. Blankets should be washed in the spring rather than the autumn, and put away in bags, always leaving out enough for the cold nights that occur even in summer. They can be kept safe and neat in pillow cases, always within reach when needed. The windows of a store-room or closet should be protected against moths and flies by a fine netting. A good way to discover the presence of moths, and also to destroy them, is to place a lighted candle in a basin of water; the moths will be attracted by the flame, and will drop into the water. The burning of camphor gum or sulphur will destroy insects. The basin of water is always necessary as a safeguard against fire. In that place your little iron pot, half filled with ashes, and the camphor or sulphur. Saturate this well with alcohol, and set it afire. Have the room closed tightly while the smoking is going on, and be sure that no one inhales the fumes. But after all these precautions, one moth may find its way into the closet or chest, and the close bag or wrapper is the only safeguard.

